# Hereditary and environmental epidemiology of sarcomas

**DOI:** 10.1186/2045-3329-2-13

**Published:** 2012-10-04

**Authors:** David M Thomas, Sharon A Savage, Gareth L Bond

**Affiliations:** 1 Division of Cancer Medicine, Sir Peter MacCallum Department of Oncology, University of Melbourne, East Melbourne, Australia; 2 Clinical Genetics Branch, Division of Cancer Epidemiology and Genetics, National Cancer Institute, National Institutes of Health, Rockville, MD, 20892, USA; 3 Ludwig Institute for Cancer Research, University of Oxford, Nuffield Department of Clinical Medicine, Old Road Campus Research Building, Oxford, OX3 7DQ, UK; 4 Sarcoma Genomics and Genetics Laboratory, Division of Cancer Medicine and Research Division, Peter MacCallum Cancer Centre, St Andrew’s Place, East Melbourne, VIC 3002, Australia

## 

Epidemiology is the study of the patterns and determinants of health-related events and the application of this knowledge to the control of disease. It is important in understanding disease incidence and helpful for the discovery of disease risk factors. Sarcomas are rare and heterogeneous and, as such, pose significant challenges epidemiologically. In most parts of the world, sarcomas are estimated to account for 1-3% of all cancers. Moreover, sarcomas comprise over 50 different histological subtypes, which are being reclassified continually, as new entities are defined with new technologies and new classifications. Sarcoma subtypes range from diseases that arise in a putative stem cell-like population (primitive neuroectodermal tumors) to those arising from morphologically recognisable connective tissues such as bone, fat and cartilage. The outcomes for patients with sarcomas are equally heterogeneous. Taking all of the subtypes together, sarcomas collectively meet the US Rare Diseases Act (2002) definition of any condition that affects fewer than 200,000 people by some margin. The peculiarities of the epidemiology of sarcoma are outlined in this series by Burningham et al. [1].

Readers of this series will be struck by the degree to which familial sarcoma syndromes have broadly contributed to understanding of cancer biology, including the fundamentals of cell cycle regulation and maintenance of the genome integrity. For example, sarcomas comprise the single most common cancer type seen in families with Li-Fraumeni syndrome, which is most commonly caused by germline mutations in *TP53.* The study of *TP53* biology in sarcomas has contributed to our knowledge of this crucial tumour suppressor that is the most somatically mutated gene in human cancers. The story of p53 demonstrates the contribution of mouse models of human disease (see papers by Ng et al. [2]; and Post [3]). Individuals with retinoblastoma, an inherited cancer syndrome, have a 500-fold increased incidence of bone sarcomas, predominately osteosarcoma (reviewed here by Kleinerman et al. [4]). Long before the gene responsible for this syndrome (*RB1*) was identified, epidemiologic studies of affected families led to the ‘two-hit’ hypothesis of tumor suppressor genes [5]. Other important examples of the hereditary sarcomas described in this series include gastrointestinal stromal tumors [6] and malignant peripheral nerve sheath tumors [7].

Despite (or perhaps because of) being rare, sarcomas have acted as sentinel events that are epidemiologically noteworthy. While it is sometimes difficult to distinguish sporadic from hereditary breast cancer, a sarcoma occurring twice within the one family is epidemiologically striking. Another feature of sarcomas is the relatively young age of onset. Cancer is generally a disease of ageing: the average age of cancer diagnosis in Western countries is in the late 60s. By contrast, sarcomas affect younger populations, while some sarcomas (primarily rhabdomyosarcoma, osteosarcoma, and Ewing sarcoma) are essentially diseases of childhood and adolescence. Epidemiologically, early onset cancer usually suggests predisposing factors are at play, whether environmental or hereditary. Environmental causes of sarcoma are less well defined, but an obvious example is the carcinogenic effects of ionising radiation (see review by Berrington de Gonzalez et al. [8]). Other potential environmental carcinogens, such as herbicides, polyvinyl chloride, arsenicals, have been linked to sarcoma incidence.

Cancer is fundamentally a genetic disease. While genome-wide expression, miRNA and copy number technologies have improved disease classification, novel sequencing approaches are likely to reform our understanding of the germline and somatic causes of cancer. These advances have the potential to better apply targeted therapies, thus further “personalizing” cancer therapy. Many somatic mutations will be ‘actionable’, a term used to define a known link between the mutation and response (or not) to the increasing range of targeted and re-purposed therapies available in the clinic. Sarcomas such as gastrointestinal stromal tumor and dermatofibrosarcoma protuberans demonstrate the power of linking genetic mutations to effective targeted therapy. While such therapeutic opportunities are immensely exciting, in general they turn cancer from an inexorably lethal disease into a chronic (and socially expensive) condition.

For most cancers, and especially for sarcomas, surgery remains critical to cure. However, once disseminated, surgery has a limited role to play in achieving cure. Detection of cancers at an early stage, prior to spread, is essential to enable effective surgery. Arguably therefore, the most effective mechanism for improved survival lies in prevention and early detection. This has already been demonstrated by the application of screening procedures in populations affected by lung, bowel and breast cancers [9]. Screening for sarcomas is not feasible in the general population due to their rarity. In certain familial cancer syndromes, such as Li-Fraumeni syndrome or Rothmund Thomson syndrome, where there is a high risk of sarcoma, this may be warranted, although further studies of the impact on clinical outcomes are needed.

The cost and benefit of screening is therefore optimised if they are applied to high-risk populations—which is where the genomic revolution comes in. Let us take breast and ovarian cancer as the best-studied basis for estimates of heritable cancer risk. Using family history alone, it is estimated that only about 5-10% of cancer has a hereditary component. Moreover, in only about half of these cases has a genetic basis been identified, usually by serially assaying for likely causal genes (eg, mutations in *BRCA1 or BRCA2* in families with lots of breast cancer). Typically, once a mutation is identified, no further testing is applied, because it is assumed that a single mutation underlies the familial predisposition. This approach has been used to generate estimates of the likely penetrance (and therefore clinical significance) of the linked mutation. By definition, de novo mutations will not be identified using familial pedigree as a screening tool, even though the clinical significance of the mutation for the affected individual and their progeny may be identical to that of an individual who inherits the same mutation from a parent.

It is highly likely that this paradigm will shift dramatically in the coming years. A recent study by Walsh and colleagues [10] applied massively parallel sequencing of 21 genes in a population of 360 patients with ovarian, peritoneal and fallopian tube cancers—unselected for family or personal history of cancer. The genes were chosen because they had been linked previously to cancer predisposition. Remarkably 82 of the 360 (24%) carried 85 pathogenic mutations in their germline. These mutations were identified as unambiguously of clinical importance. Moreover, 3 (4%) carried two such mutations. In many cases, the mutations were identified in individuals who had no family or personal history of cancer, consistent with either a striking de novo mutation rate or differences in penetrance in this cohort. Taken together, studies such as this suggest that the systematic application of massively parallel sequencing to populations with sarcoma will identify a very significant proportion who are at significantly greater risk for second cancers, or who may pass on the mutation to their offspring. In some cases, the mutation may affect the choice of therapy for the primary cancer. Clinicians may elect, for example, to avoid carcinogenic therapies in individuals carrying mutations in DNA repair genes.

This brings us back to the epidemiology of sarcomas, our understanding of their multi-factorial etiologies, and the future for our patients with these cancers. In the coming years, deep sequencing technologies may make the complex outbred genetics of human populations a tractable system to complement animal models. The application of new genetic tools may not only result in reclassification of sarcoma, but may also systematically identify people who carry germline and/or somatic mutations in cancer-causing genes. Because each cancer seems to have its own spectrum of mutations, the study of sarcomas may shed interesting light on the biology of cancer in general, above that which will be had from sequencing more common cancer types through global enterprises such as the International Cancer Genome Consortium. Because sarcomas affect a younger population, with a higher rate of known familial syndromes, it is also likely that systematic studies such as that by Walsh et al. [10] will identify a significant proportion of individuals who carry mutations in new or known ‘sarcoma genes’. The ultimate goal of epidemiology is to enable improvements in cancer outcomes. The identification of high-risk will form an essential component of screening and prevention strategies, can be used to facilitate reproductive decision making in a young population, and may affect treatment choices directly. The implications are broad, and go beyond simply identifying that risk: but it’s a start.
